# Zeolites go to mesopores with 36-ring

**DOI:** 10.1093/nsr/nwag585

**Published:** 2026-09-12

**Authors:** Ming-Hui Sun, Zi-Chen Zheng, Bao-Lian Su, Li-Hua Chen

**Affiliations:** State Key Laboratory of Advanced Technology for Materials Synthesis and Processing, Wuhan University of Technology, China; Laboratory of Inorganic Materials Chemistry (CMI), University of Namur, Belgium; State Key Laboratory of Advanced Technology for Materials Synthesis and Processing, Wuhan University of Technology, China; Laboratory of Inorganic Materials Chemistry (CMI), University of Namur, Belgium; State Key Laboratory of Advanced Technology for Materials Synthesis and Processing, Wuhan University of Technology, China

Zeolites are essential in petroleum refining and fine chemical production due to their micropores and high thermal stability. However, most industrial zeolites are 10- or 12-ring frameworks with apertures below 0.74 nm, severely limiting the diffusion of bulky molecules. Even recently reported 22-ring zeolites remain microporous [1,2]. In contrast, mesoporous materials such as Mobil Composition of Matter No. 41 (MCM-41) provide large pores but lack thermal stability and efficient active sites due to amorphous frameworks [3]. Thus, designing stable crystalline zeolites with intrinsically ordered mesopores remains a major challenge.

Li’s research group at Nanjing University reported NJU120-6, a fully crystalline mesoporous zeolite featuring 36-ring windows, which represents the largest pore aperture documented to date [4]. Its window size of a 2.571nm × 1.912 nm aperture (Fig. 1a–d) extends zeolitic structures into the mesoporous range. The framework features 36-ring channels along the *c*-axis, confirmed by interactive differential phase contrast scanning transmission electron microscopy (iDPC-STEM, Fig. 1e). Unlike the elongated channels of other mesoporous zeolites, including ITQ-37 and ITQ-43 from the Instituto de Tecnología Química (ITQ) series and ZMQ-1 (Zeolitic Materials, Qingdao Institute of Bioenergy and Bioprocess Technology, no. 1) [5–8], NJU120-6 possesses nearly circular pores, which facilitate faster diffusion and improved mass transport. The key breakthrough of this work lies in the innovative design of organic structure-directing agents (OSDAs). The authors employed bulky bisphosphonium dicationic species as OSDAs, which were fabricated into templating agents with tunable dimensions via a modular ‘Lego-like’ assembly strategy (Fig. 1f).

**Figure 1.**
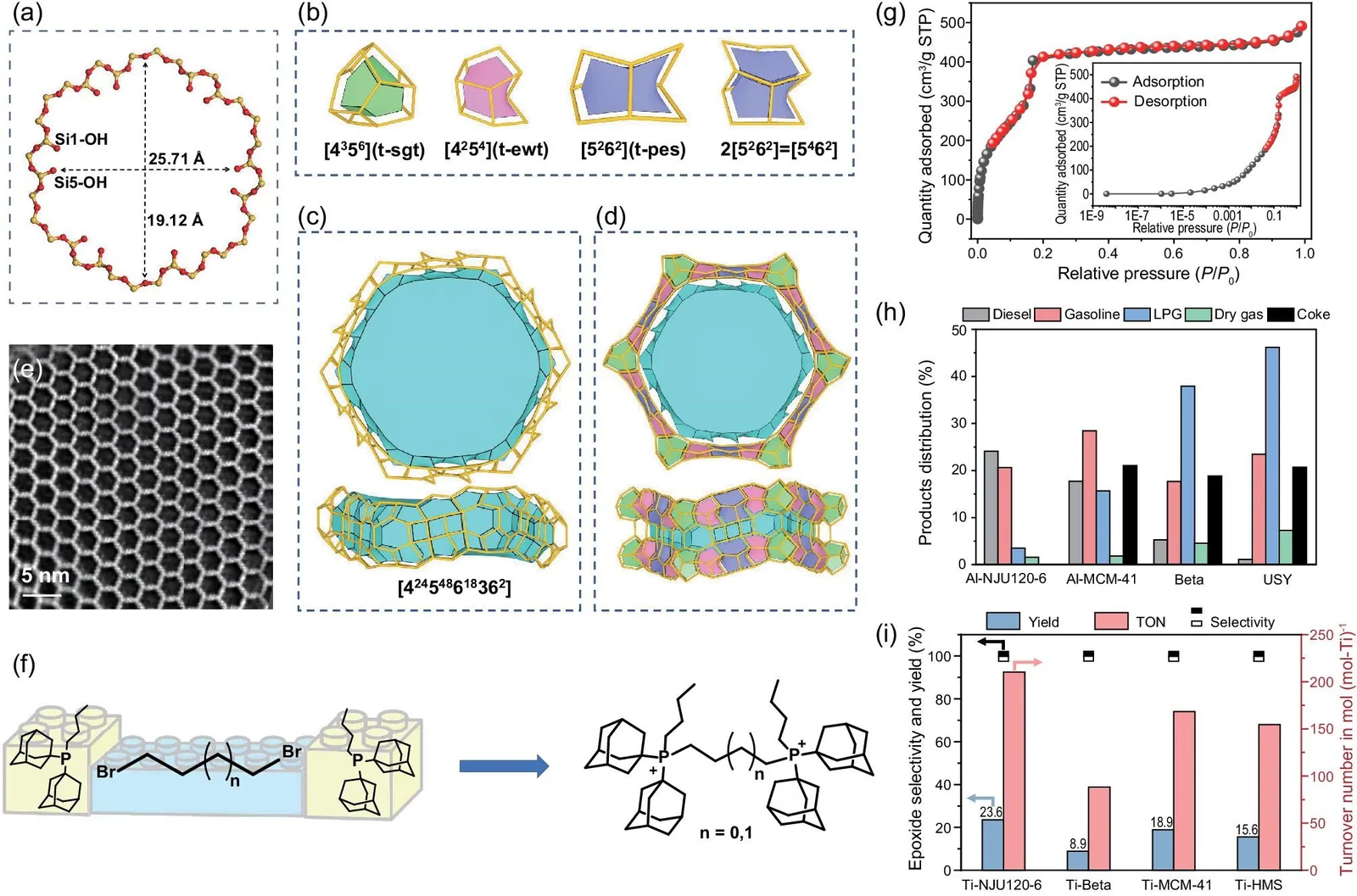
(a) Crystallographic pore apertures of NJU120-6. (b and c) Four different cages in NJU120-6 shown as solid tiles. (d) 3D pore structure in NJU120-6 with the cages embedded in the scaffolds. (e) iDPC-STEM images of calcined NJU120-6. (f) OSDA used in NJU120-6 synthesis. (g) Ar adsorption–desorption isotherm of NJU120-6 at 87 K. (h) Catalytic cracking of vacuum gas oil over Al-NJU120-6 and reference catalysts. (i) Epoxidation of cyclooctene with TBHP over Ti-NJU120-6 and reference catalysts. Adapted with permission from Li *et al*. [4].

NJU120-6 exhibits the lowest framework density among known silica zeolite, with a value of 9.39 Si atoms per nm^3^. This corresponds to a calculated material density of 0.956 g cm^−3^, notably lower than that of water. Despite its highly open framework, NJU120-6 maintains thermal stability up to at least 1173 K. After calcination and washing, Ar (87 K) and N_2_ (77 K) physisorption measurements reveal a type IV(b) isotherm (Fig. 1g), confirming the presence of mesopores. The apparent Brunauer–Emmett–Teller surface area of NJU120-6 zeolite calculated from Ar adsorption data is about 1301.9 m^2^ g^−1^, with a record-high zeolite pore volume of 0.66 cm^3^ g^−1^ using nonlocal density functional theory method.

NJU120-6 can not only incorporate aluminum over a wide Si/Al ratio range to generate Brønsted acid sites, but also introduce titanium into the framework via a one-pot method to create active sites for oxidation reactions. In the catalytic cracking of vacuum gas oil, Al-NJU120-6 achieves outstanding performance with high gasoline and diesel selectivity (44.7%), minimal gaseous byproducts and negligible coke formation, significantly outperforming USY, Beta and Al-MCM-41 (Fig. 1h). This is attributed to its weak and uniform acidity combined with ultra-large ordered mesopores, which suppresses excessive cracking and coke deposition. Similarly, Ti-NJU120-6 exhibits higher yield (23.6%) and turnover number (210.2 mol mol^−1^) in the oxidation of cyclooctene with *tert*-butyl hydroperoxide (TBHP), outperforming Ti-Beta and Ti-MCM-41 (Fig. 1i). The ultra-large, ordered pores facilitate the diffusion of bulky substrates and improve the accessibility and utilization of active sites.

NJU120-6 represents a paradigm shift in zeolite science, demonstrating that intrinsic mesoporosity can be achieved within a fully crystalline and stable framework. The modular OSDA strategy offers a versatile approach to designing zeolites with tailored pore architectures and functionalities. By bridging the gap between microporous zeolites and mesoporous materials, NJU120-6 opens new opportunities for industrial applications, including heavy oil upgrading and biomass conversion. The ability to engineer pore structures at the atomic scale brings the rational design of next-generation zeolite catalysts closer to realization. Translating NJU120-6 from a laboratory discovery to an industrial catalyst, however, faces several challenges. The current synthesis relies on bulky bisphosphonium OSDAs, which are complex and costly, underscoring the need for more economical alternatives or efficient recycling strategies to enable scalable production. Moreover, while NJU120-6 exhibits excellent thermal stability, its long-term hydrothermal stability under industrial conditions—particularly in the presence of steam and during repeated reaction–regeneration cycles—requires further improvement to ensure sustained performance. Very recently, Yu’s group reported JU-69, another 36-ring zeolite that exhibits comparable structural openness and notable hydrothermal stability, including resistance to boiling water and mild steaming [9]. When used as a support for Pd nanoparticles, JU-69 showed superior performance in the hydrogenation of bulky polycyclic aromatics, expanding the catalytic utility of such mesoporous zeolites beyond cracking and oxidation. With ongoing advances in OSDA design and synthetic optimization, extra-large-pore zeolites such as NJU120-6 and JU-69 hold strong promise for industrial processes involving bulky substrates, representing a transformative step toward next-generation heterogeneous catalysis. Beyond current applications, exploring the catalytic potential of 36-ring zeolites in other reactions is of pressing interest. In particular, introducing metal single atoms or clusters into the pore channels to enable confined catalysis for hydrogenation, dehydrogenation and redox reactions constitutes a key avenue for advancing mesoporous zeolites and expanding their catalytic utility.
